# Peer Review of “The Impact of SARS-CoV-2 Lineages (Variants) and COVID-19 Vaccination on the COVID-19 Epidemic in South Africa: Regression Study”

**DOI:** 10.2196/47384

**Published:** 2023-07-03

**Keywords:** COVID-19, infection, pandemic, vaccine, vaccination, epidemiology, transmissibility, health care, hospital admission, COVID-19 variants, SARS-CoV-2

*This is a peer-review report submitted for the paper “The Impact of SARS-CoV-2 Lineages (Variants) and COVID-19 Vaccination on the COVID-19 Epidemic in South Africa: Regression Study.”*

## Round 1 Review

### General Comments

In this article [1], the authors study the emerging variants of SARS-CoV-2 at the immune and epidemiological levels. The authors conclude that the Delta, Beta I VOC SARS-CoV-2, and lineage cluster, predominantly B.1.1.54, B.1.1.56 C.1 SA SARS-CoV-2 were observed to cause similar cases of COVID-19 hospital mortality and discharge rates in South African hospitals.

### Specific Comments

The article seems good to me but too complex and difficult to follow, it should be “lightened.”

#### Major Comments

When talking about COVID-19 and its variants, some important points should be clarified that inform and prepare the reader well to deal with the specifics. Therefore, to make this paper more complete and interesting for the readers of this important journal, the authors should expand a bit of the discussion on cytokines. On this subject, three important articles have recently been reported. Below I list these interesting articles that should be studied, incorporated into the meaning, and reported briefly in the discussion and in the list of references.

- Conti P, Caraffa A, Tetè G, Gallenga CE, Ross R, Kritas SK, et al. Mast cells activated by SARS-CoV-2 release histamine which increases IL-1 levels causing cytokine storm and inflammatory reaction in COVID-19. *J Biol Regul Homeost Agents*. 2020;34(5):1629-1632. PMID:32945158 doi:10.23812/20-2EDIT
- Ronconi G, Teté G, Kritas SK, Gallenga CE, Caraffa A, Ross R, et al. SARS-CoV-2, which induces COVID-19, causes kawasaki-like disease in children: role of pro-inflammatory and anti-inflammatory cytokines. *J Biol Regul Homeost Agents*. 2020;34(3):767-773. PMID:32476380 doi:10.23812/EDITORIAL-RONCONI-E-59
- Conti P, Caraffa A, Gallenga CE, Ross R, Kritas SK, Frydas I, et al. Coronavirus-19 (SARS-CoV-2) induces acute severe lung inflammation via IL-1 causing cytokine storm in COVID-19: a promising inhibitory strategy.*J Biol Regul Homeost Agents*. 2020 Nov-Dec;34(6):1971-1975. PMID:33016027 doi:10.23812/20-1-E

#### Minor Comments

Some legends should be expanded.

I believe these suggestions are important for improving this paper. Without these corrections, the paper cannot be published. So I recommend minor revision.
